# Melatonin Reduces NLRP3 Inflammasome Activation by Increasing α7 nAChR-Mediated Autophagic Flux

**DOI:** 10.3390/antiox9121299

**Published:** 2020-12-18

**Authors:** Víctor Farré-Alins, Paloma Narros-Fernández, Alejandra Palomino-Antolín, Céline Decouty-Pérez, Ana Belen Lopez-Rodriguez, Esther Parada, Alicia Muñoz-Montero, Vanessa Gómez-Rangel, Francisco López-Muñoz, Eva Ramos, Águeda González-Rodríguez, Luis Gandía, Alejandro Romero, Javier Egea

**Affiliations:** 1 Molecular Neuroinflammation and Neuronal Plasticity Research Laboratory, Hospital Universitario Santa Cristina, Instituto de Investigación Sanitaria-Hospital Universitario de la Princesa, 28006 Madrid, Spain; victorfarre@hotmail.com (V.F.-A.); palomanf22@gmail.com (P.N.-F.); alejandra.palominoantolin@gmail.com (A.P.-A.); celinedecouty96@gmail.com (C.D.-P.); lopezrodr.ab@gmail.com (A.B.L.-R.); esther.parada@inv.uam.es (E.P.); vanessagr2014@gmail.com (V.G.-R.); 2 Instituto Teófilo Hernando, Departamento de Farmacología y Terapéutica, Facultad de Medicina, UAM, 28029 Madrid, Spain; alimunozmontero@gmail.com (A.M.-M.); luis.gandia@uam.es (L.G.); 3 Faculty of Health Sciences, University Camilo José Cela, Villanueva de la Cañada, 28692 Madrid, Spain; flopez@ucjc.edu; 4 Neuropsychopharmacology Unit, Hospital 12 de Octubre Research Institute (i+12), 28041 Madrid, Spain; 5 Portucalense Institute of Neuropsychology and Cognitive and Behavioural Neurosciences (INPP), Portucalense University, 4200-072 Porto, Portugal; 6 Thematic Network for Cooperative Health Research (RETICS), Addictive Disorders Network, Health Institute Carlos III, MICINN and FEDER, 28029 Madrid, Spain; 7 Department of Pharmacology and Toxicology, Faculty of Veterinary Medicine, Complutense University of Madrid, 28040 Madrid, Spain; eva.ramos@ucm.es (E.R.); manarome@ucm.es (A.R.); 8 Research Unit, Hospital Universitario Santa Cristina, Instituto de Investigación Sanitaria-Hospital Universitario de la Princesa, 28006 Madrid, Spain; aguedagr.phd@gmail.com; 9 Centro de Investigación Biomédica en Red de Enfermedades Hepáticas y Digestivas (CIBEREHD, ISCIII), 28029 Madrid, Spain

**Keywords:** melatonin, inflammasome, α7 nicotinic receptor, autophagy

## Abstract

Microglia controls the immune system response in the brain. Specifically, the activation and dysregulation of the NLRP3 inflammasome is responsible for the initiation of the inflammatory process through IL-1β and IL-18 release. In this work, we have focused on studying the effect of melatonin on the regulation of the NLRP3 inflammasome through α7 nicotinic receptor (nAChR) and its relationship with autophagy. For this purpose, we have used pharmacological and genetic approaches in lipopolysaccharide (LPS)-induced inflammation models in both in vitro and in vivo models. In the BV2 cell line, LPS inhibited autophagy, which increased NLRP3 protein levels. However, melatonin promoted an increase in the autophagic flux. Treatment of glial cultures from wild-type (WT) mice with LPS followed by extracellular adenosine triphosphate (ATP) produced the release of IL-1β, which was reversed by melatonin pretreatment. In cultures from α7 nAChR knock-out (KO) mice, melatonin did not reduce IL-1β release. Furthermore, melatonin decreased the expression of inflammasome components and reactive oxygen species (ROS) induced by LPS; co-incubation of melatonin with α-bungarotoxin (α-bgt) or luzindole abolished the anti-inflammatory and antioxidant effects. In vivo, melatonin reverted LPS-induced cognitive decline, reduced NLRP3 levels and promoted autophagic flux in the hippocampi of WT mice, whereas in α7 nAChR KO mice melatonin effect was not observed. These results suggest that melatonin may modulate the complex interplay between α7 nAChR and autophagy signaling.

## 1. Introduction

Inflammation is a crucial signal response to initiate protective immunity. However, uncontrolled inflammation can be harmful, leading to tissue damage and the development of inflammatory and/or autoimmune diseases [1]. The initiation of inflammation depends on pattern-recognition receptors (PRRs), such as Toll-like receptor 4 (TLR4), which recognizes pathogen-associated molecular patterns (PAMPs) or danger-associated molecular patterns (DAMPs), also termed alarmins or damaged cell-derived molecules, leading to the production of inflammatory cytokines such as IL-1β and TNF-α [2,3]. In the central nervous system (CNS), nucleotide-binding and oligomerization domain (NOD)-like receptor pyrin domain containing 3 (NLRP3) inflammasome has gained attention because of its abundance in the brain and its ability to detect different damage signals, including reactive oxygen species (ROS), mitochondrial DNA (mtDNA), extracellular adenosine triphosphate (ATP) and components of dying cells [4,5]. Inflammasomes are multi-protein molecular platforms involved in the activation of caspase-1, followed by a downstream cleavage and the release of pro-inflammatory cytokines, such as IL-1β and IL-18, from innate immune cells [5,6]. In this regard, hyperinflammation caused by uncontrolled inflammasome activation is linked with many inflammatory, metabolic, degenerative and aging-related diseases [7]. Therefore, the increasing interest in knowing the mechanisms that regulate the inflammatory pathways led to carefully investigate and explore its relevance as a valuable strategy for the treatment of inflammation. Accumulating evidence indicates that NLRP3 and autophagy pathways are linked by reciprocal regulation, and recent studies have shown autophagy-dependent degradation of NLRP3 [8,9,10,11,12], pointing to autophagy as a main regulatory mechanism of inflammasomes [13].

Microglia represent the major cellular component of the innate immune system of the brain. Microglial cells are considered the earliest responders to environmental changes, including pathological insults, by clearing debris and restoring homeostasis in the CNS [14]. Moreover, under non-inflammatory conditions, they also maintain tissue integrity [15,16]. Microglia express different PRRs that are responsible for the early recognition of PAMPs and DAMPs, such as TLRs, NOD-like receptors (NLRs) and the triggering receptor expression on myeloid cells 2 (TREM2) [17,18]. Recent findings have involved microglia over-activation in the initiation and maintenance of inflammatory responses in the context of infectious brain diseases, acute CNS injury and several neurodegenerative diseases [19,20]. 

Under physiological conditions, circulating melatonin (*N*-acetyl-5-methoxytryptamine) is mainly released from the pineal gland. However, its production has also been demonstrated in several extrapineal organs, including immune system cells, CNS, bone marrow, skin and likely other tissues [21]. The therapeutic value of melatonin in mental disorders and neurodegenerative diseases, cardiovascular diseases, cancer, gastrointestinal pathologies or infectious diseases has been highlighted in numerous scientific reports [22,23,24]. The multiple actions of melatonin have been attributed to its potent antioxidant activity, modifying energy metabolism as well as its ability to modulate the immune response and neuroinflammation among others [25,26,27,28]. In this respect, the immunomodulatory actions of melatonin at acute high doses may counteract the response to sepsis [29,30]. However, many melatonin actions are also mediated through its interaction with two high-affinity G-protein-coupled receptors, named MT1 and MT2, whose physiological functions and pharmacological properties are well documented [28]. 

In previous studies, we have demonstrated that melatonin provides a protective effect in brain ischemia that, at least in part, depends on the nicotinic receptor (nAChR) activation, the overexpression of HO-1 and the reduction of oxidative stress [31]. Moreover, melatonin may regulate autophagic flux via α7 nAChRs [32], and activation of these receptors in microglia has a potent anti-inflammatory role [33,34]. Here, we show that melatonin reduces oxidative stress and NLRP3 triggering due to activation of autophagic flux. These effects are partially mediated through α7 nAChRs, since in α7 nAChRs knock-out mice the melatonin effect remained unchanged. 

## 2. Materials and Methods 

### 2.1. Microglia BV2 Cell Line

BV2 cells (murine microglia) were maintained in Dulbecco’s Modified Eagle Medium (DMEM) supplemented with 10% FBS and antibiotics (penicillin 100 U/mL, streptomycin 100 μg/mL). Cultures were seeded into flasks containing supplemented medium and kept at 37 °C in a humidified atmosphere of 5% CO_2_ and 95% air. For assays, BV2 cells were subcultured in 48-well plates at a seeding density of 1 × 10^5^ cells per well. Cells were treated with the drugs before confluence in RPMI with 10% FBS.

### 2.2. Mixed Glia Cultures

Mixed glial cultures were prepared from cerebral cortices of 3-day-old mice as previously described [35]. Briefly, after removing the meninges and blood vessels, the forebrains were gently dissociated by repeated pipetting in DMEM/F12 medium. After mechanical dissociation, cells were seeded in DMEM/F12 with 20% FBS at a density of 3 × 10^5^ cells/mL and cultured at 37 °C in humidified 5% CO_2_ and 95% air. Medium was replaced after 5 days in vitro (DIV) by DMEM/F12 and 10% FBS. Cultures were used at confluence, which was achieved after 10–12 DIV.

### 2.3. Reagents

Cultures were treated with lipopolysaccharide (LPS) (1 µg/mL) and ATP (5 mM) (Sigma-Aldrich, Madrid, Spain) to create a model of NLRP3 inflammasome activation. Pharmacological treatments were melatonin (10 nM), α-bungarotoxin (α-bgt, 100 nM) and luzindole (1 µM) (Sigma-Aldrich, Madrid, Spain). The compounds used to assess autophagy were chloroquine (inhibitor; Sigma-Aldrich, Madrid, Spain) and rapamycin (inductor; Sigma-Aldrich, Madrid, Spain).

### 2.4. Griess Reaction

The NO assay was performed using the previously reported protocols [36]. Briefly, 100 μL of culture supernatants was mixed with the same volume of Griess assay regent (1% sulfanilamide, 0.1% naphthylethylenediamine dihydrochloride, and 2.5% phosphoric acid). Then, 10 min later, absorbance was measured at 560 nm.

### 2.5. ROS Measurement

To measure cellular ROS, we used the molecular probe H2DCFDA. BV2 cells were loaded with 5 µM H_2_DCFDA as previously described [35]. Fluorescence was measured in a fluorescence-inverted NIKON Eclipse TE200 microscope. Wavelengths of excitation and emission were 485 and 520 nm, respectively.

### 2.6. Determination of IL-1β Levels in the Culture Medium

After the different drug treatments, IL-1β levels were measured by using a specific ELISA kit. Supernatant samples were obtained at the indicated times and subjected to the ELISA analysis according to the recommendations of the supplier (DY401, R&D Systems, Minneapolis, MN, USA).

### 2.7. Determination of Pyroptosis

The viability of glial cultures was measured by lactate dehydrogenase (LDH) cytotoxicity assay kit (11644793001, Thermo Fisher, Madrid, Spain). In order to measure extracellular LDH activity, 50 µL of medium culture were incubated with 50 µL of the detection kit; to detect intracellular LDH, we lysed cells with 200 µL of 1% Triton X-100 (ThermoFischer, Madrid, Spain), and we incubated 50 µL with the same volume of the detection kit. After 30 min of incubation at 37 °C, LDH enzymatic activity was measured by absorbance at 490–620 nm. To determine the percentage of death cells, we used the following formula: extracellular LDH activity/(extracellular LDH activity + intracellular LDH activity).

### 2.8. Animals

Animal experiments were conducted in accordance with the ARRIVE guidelines, the International Council for Laboratory Animal Science, and the European Union 2010/63/EU Guidelines. The experimental protocol was approved by the Institutional Ethics Committee of Autonomous University of Madrid (Spain). Experiments were performed on 3-month male C57BL/6J wild-type and α7 nAChR knockout mice (25–30 g weight). Animals were group-housed in controlled temperature under a 12 h light/dark cycle with ad libitum access to food and water. Every effort was made to minimize the number of animals used and their suffering.

### 2.9. Novel Object Recognition Test in Mice

Novel object recognition (NOR) test is a commonly used behavioral test for assessing recognition memory in mice [37]. Animals were placed for 10 min on a field (40 × 40 × 40 cm made up of polyvinyl chloride) during 3 consecutive days. On the first day (T0), mice explored the empty box. On the second day (T1), animals were placed on the field with two identical objects (cylindrical glass bottles, heavy enough to prevent mice from moving; height, 22 cm; diameter, 9 cm) and they were allowed to explore them for 10 min. On the third day (T2), a new object (novel object) was placed on the site of one of the old objects (familiar object) and the other one was maintained. Exploration of the objects was timed with stopwatches when animals sniffed at, whisked at, or looked at the objects from no more than 2 cm away. All locations for the objects were counterbalanced among groups, and objects and the NOR field was cleaned with 0.1% acetic acid between trials to equate olfactory cues. The amount of time spent investigating the novel or familiar object was video recorded for 10 min and evaluated by a blinded observer. NOR is based in the assumption that rodents have innate preference for new objects, so mice that remember familiar objects will spend more time exploring the new object. Discrimination index in the T2 was estimated as follows: Discrimination Index = [Time exploring novel object − Time exploring familiar object)/(Time exploring novel object + Time exploring familiar object)] × 100% [38]. LPS (250 μg/kg) was injected intraperitoneally (i.p.) immediately after the end of T1 phase. Melatonin (10 mg/kg i.p.) was administered immediately after the end of T0 and T1 phase.

### 2.10. Immunoblotting and Image Analysis

After the different treatments, BV2 microglial cells or hippocampi of mice were lysed in ice-cold lysis buffer (1% Nonidet P-40, 10% glycerol, 137 mM NaCl, 20 mM Tris-HCl, pH 7.5, 1 g/mL leupeptin, 1 mM PMSF, 20 mM NaF, 1 mM sodium pyrophosphate, and 1 mM Na3VO4). Proteins (30 µg) from the cell or hippocampi lysate were resolved by SDS–PAGE and transferred to Immobilon-P membranes (Millipore Corp., Burlington, MA, USA). Membranes were incubated with anti-NLRP3 (1:1000; AG-20B-0014, AdipoGen, San Diego, CA, USA), anti-IL-1β (1:1000; AF-401-NA, R&D Systems, Minneapolis, MN, USA), anti-LC3 (1:1000; 4108, Cell Signaling Technology, Danvers, MA, USA), anti-p62 (1:1000; PM045, MBL, Woods Hole, MA, USA) or anti-β-actin (1:50,000; A3854, Sigma-Aldrich, Madrid, Spain). Appropriate peroxidase-conjugated secondary antibodies (1:5000; Santa Cruz Biotechnology, Dallas, TX, USA) were used to detect proteins by enhanced chemiluminescence. Different band intensities corresponding to immunoblot detection of protein samples were quantified using the Scion Image program. Immunoblot images correspond to a representative experiment.

### 2.11. RNA Extraction and Quantitative Real-Time PCR

Total mRNA from BV2 cells and hippocampus of wild-type (WT) and α7 nAChR KO mice was obtained by TRIzol method (10296-028, Invitrogen, California, USA). cDNA was synthetized with the iScript cDNA synthesis kit (1708891, Biorad, California, USA) and qRT-PCR was performed using Power SYBR Green Master Mix (RR420L, Takara, Japan) in the QuantStudio 5 PCR system (Applied Biosystems, California, USA). The target genes and the specific primers were the following: NLRP3 (forward, 5′-TTCAATCTGTTGTTCAGCTC-3′; reverse, 5’-TTCAATCTGTTGTTCAGCTC-3′), pro-caspase-1 (forward, 5′-GGGACATTAAACGATTAAACAAGAATCC-3′; reverse, 5′-GGAAGTATTGGCTTCTTATTGG-3′), pro-IL-1β (forward, 5′-GAAGAGCCCATCCTCTGTGA; reverse, 5′-TTCATCTCGGAGCCTGTAG). Data were normalized to the expression of the housekeeping gene B2M and calculated by the Δ/Δ Ct method.

### 2.12. Immunofluorescence and Confocal Imaging

Mice were anesthetized with ketamine (100 mg/kg i.p.)/xylazine (10 mg/kg i.p.) and perfused intracardially with PBS. Brains were removed and fixed in 4% paraformaldehyde overnight, followed by a 24 h immersion in sucrose 30%. Then, they were embedded in Optimal Cutting Temperature (OCT) compound, frozen at −80 °C and cut with a cryostat (CM 1100; Leica Microsystems, Wetzlar, Germany) at 16 μm thickness. Sections were incubated overnight at 4 °C with the primary antibody anti-p62 (1:1000; PM045, MBL, Massachusetts, USA) diluted in PBS with 0.3% Triton X-100 and 10% of normal goat serum. After three washes with PBS, slices were incubated with secondary antibodies Alexa Fluor 568-conjugated rabbit IgGs (1:500; Invitrogen, Carlsbad, CA, USA) for 1 h and nuclei were stained with DAPI (1:1000, D1306, ThermoFisher, Madrid, Spain). The slices were coverslipped with the mounting medium Fluoromount-G (0100-01, SouthernBiotech, Birmingham, AL, USA). Immunofluorescence images were obtained at 40× from CA1 regions of the hippocampus using the confocal microscope Leica TCS-SP5 (Leica Microsystems, Wetzlar, Germany). Images were processed with the program ImageJ 1.52e (ImageJ, RRID:SCR_003070).

### 2.13. Statistical Evaluation

Data were presented as mean ± SD. Differences between groups were determined by applying two-tailed t-test, one-way ANOVA followed by Tukey’s post-hoc or two-way ANOVA followed by Tukey’s post-hoc when appropriate. A *p* < 0.05 was considered statistically significant. All statistical analysis and graphical representation were performed using GraphPad Prism 8.0 software program (GraphPad Software Inc., RRID: SCR_002798).

## 3. Results

### 3.1. LPS Inhibits Autophagic Flux

**Figure 1:**
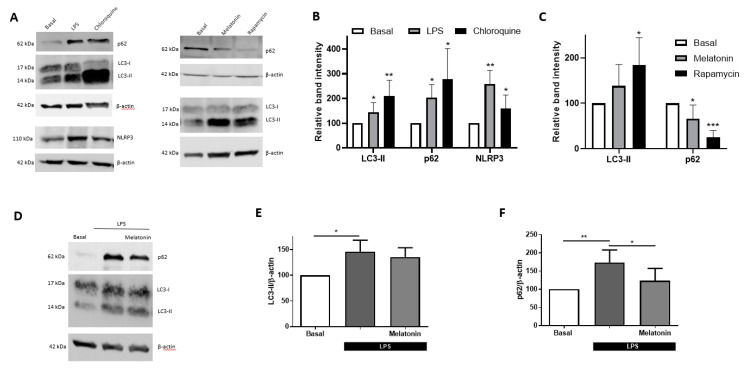
Autophagy and NLRP3 measurements in BV2 cultures. (**A**) Representative immunoblots of p62, LC3 and NLRP3 expression. (**B**) Analysis of p62, LC3-II and NLRP3 in BV2 cells treated with lipopolysaccharide (LPS) (1 µg/mL) or chloroquine (50 nM) for 4 h (*n* = 5). * *p* < 0.05, ** *p* < 0.01 vs. Basal cells. Unpaired t test. (**C**) Analysis of p62 and LC3-II, in cells incubated with melatonin (10 nM) or rapamycin (27 nM) for 24 h. * *p* < 0.05, *** *p* < 0.001 vs. Basal cells. Unpaired *t*-test. (**D**) Representative immunoblot of p62 and LC3 expression in BV2 cells. Analysis of (**E**) LC3-II and (**F**) p62 in 24 h pretreated cells with or without melatonin (10 nM) followed by incubation with LPS (1 µg/mL) during 4 h. * *p* < 0.05 and ** *p* < 0.01 vs. LPS-treated cells. One-way ANOVA test with Tukey’s multiple comparisons test. Data of all experiments are represented as mean ± SD.

Given all the above information, it seems reasonable to explore the joint-regulation of autophagy and inflammation. It has been shown recently that LPS blocks autophagy in N9 microglial cells [39]. Thus, first of all, we wanted to know if using an inflammatory stimulus, such as LPS, may inhibit autophagic flux in microglial BV2 cells. For this purpose, BV2 cells were treated with LPS (1 µg/mL) for 4 h and, at the end of the experiment, we analyzed the expression of two key proteins in the autophagic process, LC3-II and p62. LPS increased the expression of LC3-II and p62 by 45% and 103% compared to control levels, respectively (Figure 1A,B). Chloroquine (50 nM), an autophagy inhibitor, produced the same effect than LPS, although the rise was slightly higher (Figure 1A,B). These results suggest that LPS blocks autophagic flux in a similar way to chloroquine, confirming that acute inflammation induces the blockage of autophagy.

### 3.2. Melatonin Restores LPS-Blocked Autophagic Flux 

Melatonin is a versatile neuroprotective agent in different disease models. The protective effects of melatonin are related to its direct ROS scavenging and anti-oxidative stress actions [40]. In this context, Chen et al. [41] demonstrated that melatonin protects against subarachnoid hemorrhage by increasing autophagy. Based on this evidence, we aimed to assess the potential effect of melatonin alone in autophagy. Hence, BV2 cultures were stimulated for 24 h with melatonin (10 nM) or rapamycin (27 nM). Rapamycin, an autophagy inductor, produced a significant increase in LC3-II and a decrease in p62, while melatonin was able to significantly reduce p62 levels (Figure 1A,C). Then, we evaluated the effect of melatonin in LPS-treated microglial cells. Pretreatment of 24 h with melatonin (10 nM) followed by 4 h of LPS (1 µg/mL) did not modify LC3-II levels, whereas the protein levels of p62 were significantly reduced (Figure 1D–F), which indicates that melatonin has the ability to activate the autophagic flux. 

### 3.3. Melatonin Inhibits IL-1β Release and Pyroptosis in the In Vitro Model of Inflammasome Activation in Mixed Glial Cultures

**Figure 2:**
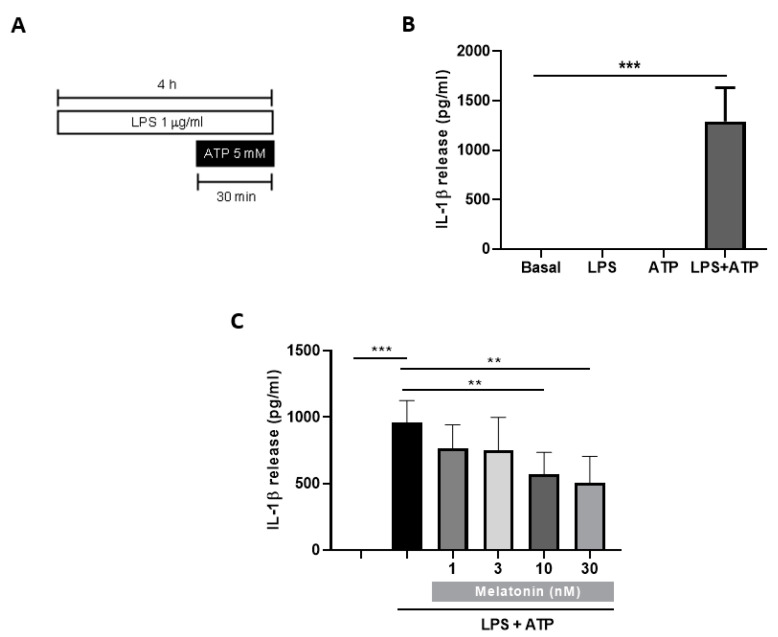
IL-1β release and cell death in glial cultures stimulated with LPS and extracellular adenosine triphosphate (ATP). (**A**) Protocol used to induce IL-1β release. Mixed glial cultures were treated with LPS (1 µg/mL) for 4 h and ATP (5 mM) during the last 30 min. (**B**) IL-1β release (measured by ELISA) of cell cultures treated with LPS for 4 h, ATP for 30 min, or a combination of both stimuli (*n* = 4). (**C**) IL-1 β measurement in cell cultures pretreated with melatonin (1, 3, 10, 30 nM) prior to LPS (1 µg/mL) plus ATP (5 mM) stimulation (*n* = 5). *** *p* < 0.001 and ** *p* < 0.01 vs. LPS+ATP. One-way ANOVA with Tukey’s multiple comparisons test. Data of all experiments are represented as mean ± SD.

Once we have validated the relationship between inflammation and autophagy in an LPS-treated BV2 cells model, we wanted to deepen in the innate immune system defense against inflammation. In recent years, a growing number of studies have focused on the relationship between autophagy and the NLRP3 inflammasome. In Figure 1B, we show that the inhibition of the autophagic process with chloroquine significantly elevated NLRP3 proteins levels compared to the control group. To continue exploring the involvement of the NLRP3 inflammasome, we measured IL-1β release in glial cultures using the protocol shown in Figure 2A. We used LPS for 4 h to increase the expression of NLRP3 inflammasome components, a process called “priming”. In the last 30 min we added ATP, which enabled NLRP3 oligomerization and activation and, finally, the release of IL-1β to the culture medium. Stimulation of cultures from WT mice with LPS (1 µg/mL) during 4 h plus the addition of ATP (5 mM) for the last 30 min produced the release of large amounts of IL-1β (Figure 2B). Both stimuli (LPS and ATP) were added on their own to the medium and they did not cause any effect (Figure 2B), indicating that the two consecutive stimuli are necessary to activate NLRP3-induced IL-1β release. 

In this model, we next evaluated the effect of nanomolar concentrations of melatonin (1, 3, 10, 30 nM) on IL-1β release. Figure 2C shows that pretreatment of 24 h with melatonin was able to significantly reduce the levels of the cytokine at 10 and 30 nM. However, we have no effect when melatonin was co-incubated together LPS (data not shown). Thus, we used melatonin at 10 nM to study its effects on inflammasome activation. 

### 3.4. Melatonin Effects Are Mediated by α7 nAChRs in Microglia Cells

**Figure 3:**
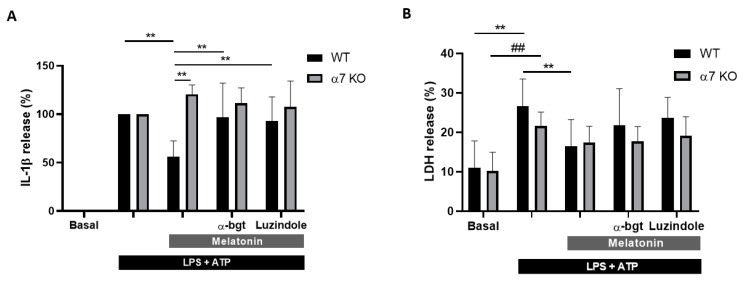
IL-1β release and cell death analyses in mixed glial cultures obtained from WT and α7 nAChRs knock-out (KO) mice stimulated with LPS and ATP (see protocol in Figure 2A). Melatonin (10 nM), α-bungarotoxin (α-bgt, 100 nM) and luzindole (1 μM) were added to the medium 24 h before stimulation with LPS (1 µg/mL). (**A**) IL-1β release of treated glial cultures from wild-type (WT) (*n* = 7) and α7 nAChRs KO (*n* = 5) mice. LPS+ATP values were considered the maximum release (100%). ** *p* < 0.01 vs. Melatonin WT. (**B**) Cell death analysis of treated glial cultures from WT (*n* = 7) and α7 nAChRs KO (*n* = 7) mice. ** *p* < 0.01 vs. LPS+ATP WT, ## *p* < 0.01 vs. LPS+ATP α7 KO. Two-way ANOVA with Tukey’s multiple comparisons test. Data of all experiments are represented as mean ± SD.

In the inflammasome activation model we evaluated whether α7 nAChRs are involved in initiating the cellular response produced by melatonin. For this purpose, we evaluated the role of α7 nAChRs using a genetic model (α7 nAChRs KO mice) and the pharmacological blockage of the receptor with α-bgt (100 nM), which acts as a selective antagonist of this receptor. In glial cultures from WT mice, melatonin (10 nM) reduced IL-1β release by 44%, whereas the co-treatment with α-bgt significantly abolished the decrease (reduction of 15%) (Figure 3A). Luzindole (1 μM), a melatonin receptor inhibitor, also reverted the effect of melatonin on IL-1β release in cultures from WT mice (Figure 3A). However, in cultured glial cells from α7 nAChRs KO mice, melatonin did not produce any effect in the release of IL-1β (Figure 3A). LDH assay revealed that pyroptotic cell death is also affected in α7 nAChRs KO mice. In cultures of WT mice, α-bgt reverted the protection exerted by melatonin (Figure 3B); however, melatonin did not diminish cell death in cultures of α7 nAChRs KO mice (Figure 3B). These results suggest that melatonin acts through α7 nAChRs to reduce NLRP3’s inflammasome activation.

**Figure 4:**
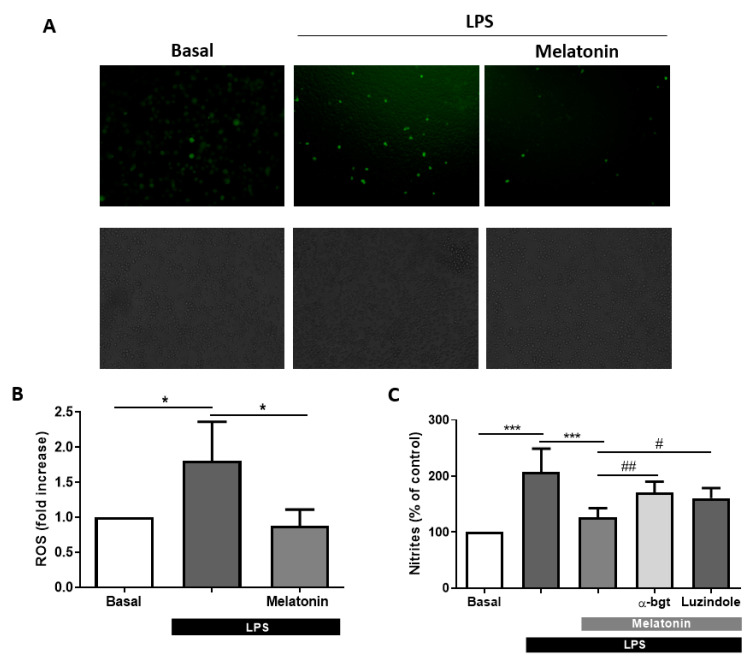
Production of reactive oxygen species (ROS) and nitrites in microglia BV2 cultures. (**A**) Images of BV2 cell cultures loaded with H2DCFDA (upper row, fluorescent images; bottom row, bright-field images of the same fields). (**B**) Cells were pretreated with melatonin (10 nM) for 24 h and then incubated with LPS (1 µg/mL) for 4 h. After the LPS-treatment, intracellular ROS production was measured by adding to the medium the fluorescent probe H2DCFDA (5 µM) (*n* = 5). (**C**) Nitrites released by BV2 determined by Griess reaction. Cells were pretreated during 24 h with melatonin (10 nM) with or without α-bgt (100 nM) and luzindole (1 μM). After that time, cultures were incubated with LPS (1 µg/mL) for 24h (*n* = 6). * *p* < 0.05 and *** *p* < 0.001 vs. LPS; # *p* < 0.05 and ## *p* < 0.01 vs. LPS + Melatonin. One-way ANOVA with Tukey’s multiple comparisons test. Data of all experiments are represented as mean ± SD.

Notwithstanding the above, melatonin presents several antioxidant effects that afford protection against cellular stress [25]. In this research, we hypothesized that melatonin could reduce oxidative stress in microglia. For the achievement of the purposes, we used BV2 microglial cells which were stimulated during 4 h with LPS (1 µg/mL), and intracellular ROS production was measured by the fluorescent probe H2DCFDA. Previously, cultures were pre-treated with melatonin (10 nM) for 24 h. After the specified time, we took representative fluorescent images of every culture that were analyzed to determine the intensity of fluorescence and, consequently, the burden of intracellular ROS. LPS treatment increased the production of ROS in BV2 cell cultures by 1.8-fold, whereas melatonin totally abolished LPS-induced ROS generation (Figure 4A,B). Moreover, to evaluate another parameter related to oxidative stress and cellular damage, we tested the release of nitrites by Griess reaction. Stimulation of microglial cultures with LPS (1 µg/mL) during 24 h produced the release of nitrites in the culture medium (107% of increase compared to non-treated cells), while melatonin (10 nM) lowered the levels of nitrites release (126%) compared to control levels. The antioxidant effect was lost when we administered α-bgt (100 nM) and luzindole (1 μM) to the cultures, rising nitrites levels by 170% (Figure 4C). Considering these results, we have observed a relationship between the reduction of IL-1β and oxidative stress levels after 4 h of the inflammatory stimulus; additionally, melatonin was not able to reduce neither IL-1β release nor nitrites production when α7 nAChRs were blocked.

**Figure 5:**
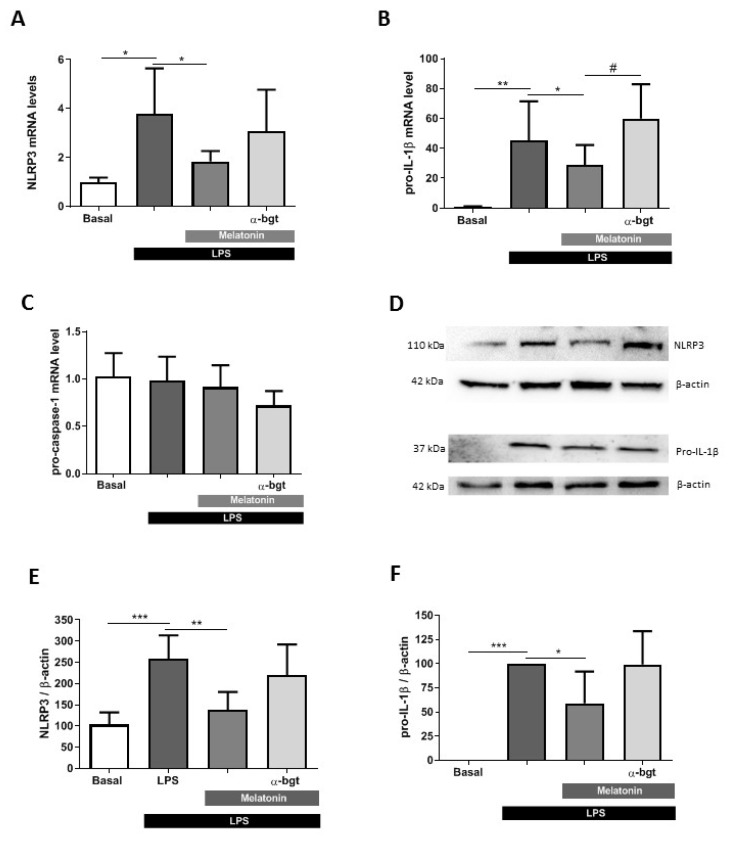
Effect of melatonin on inflammasome-related genes and proteins in microglia BV2 cells. mRNA levels of (**A**) NLRP3 (*n* = 7), (**B**) pro-IL-1β (*n* = 7) and (**C**) pro-caspase-1 (*n* = 5) in BV2 cultures treated with LPS (1 µg/mL) during 4 h in the presence or absence of melatonin (10 nM) and α-bgt (100 nM) during the previous 24 h. (**D**) Representative immunoblot of NLRP3 and pro-IL-1β expression in BV2 samples. Quantification of (**E**) NLRP3 (*n* = 5) and (**F**) pro-IL-1β (*n* = 6) protein expression after 4 h LPS (1 µg/mL) treatment in microglial cultures pretreated or not with melatonin (10 nM) and α-bgt (100 nM) during 24 h. * *p* < 0.05, ** *p* < 0.01, *** *p* < 0.001, vs. LPS; # *p* < 0.05 vs. LPS + Melatonin. One-way ANOVA with Tukey’s multiple comparisons test. Data of all experiments are represented as mean ± SD.

Once we demonstrated that melatonin modifies IL-1β release and may be related to the decrease of ROS levels, we decided to explore the genetic expression and protein levels of NLRP3 inflammasome components. We analyzed the changes in NLRP3, pro-IL-1β and pro-caspase-1 gene expression after the treatment of BV2 cells with LPS (1 µg/mL) during 4 h. Previously, cells were incubated for 24 h with melatonin (10 nM) in the presence or absence of α-bgt (100 nM). qRT-PCR analysis revealed that LPS increased by 3.7- and 4.5-fold NLRP3 and pro-IL-1β gene expression, respectively. In both cases, melatonin significantly reduced NLRP3 and pro-IL-1β gene expression, whereas α-bgt partially reverted the melatonin effect (Figure 5A,B). Regarding pro-caspase-1, we did not detect any change in gene expression (Figure 5C). We also analyzed by Western blot the inflammasome protein levels in BV2 microglia cells. NLRP3 protein levels increased above 150% compared to basal conditions (Figure 5D,E). BV2 cultures did not express pro-IL-1β in non-stimulated conditions, whereas it was strongly induced in LPS-treated cultures (Figure 5D,F). In both cases, pretreatment with melatonin (10 nM) significantly reduced the increase produced by LPS; in contrast, α-bgt (100 nM) abolished the effect induced by melatonin (Figure 5D–F). Thus, melatonin is able to decrease the expression of inflammasome proteins through α7 nAChRs in microglial cells.

### 3.5. Melatonin Treatment Improves Cognitive Deficits, Reduces Inflammasome Components Expression and Restores Autophagic Flux in Mice Through α7 nAChRs

**Figure 6:**
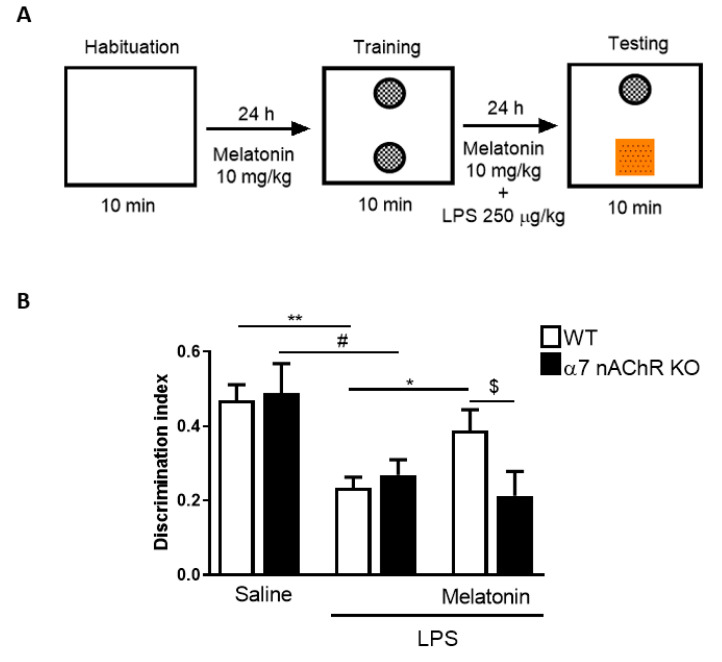
Melatonin effects on novel object recognition (NOR) test in WT and α7 nAChR KO mice. (**A**) Illustration of the protocol used to evaluate NOR test. WT and α7 nAChR KO mice were treated with melatonin (10 mg/kg, i.p.), and after 24 h were injected with LPS (250 μg/kg, i.p.). Next day mice were subjected to the test. (**B**) Discrimination index in WT and α7 nAChR KO mice in saline-treated, LPS-treated and melatonin-treated groups (*n* = 7–8 per group). * *p* < 0.05 and ** *p* < 0.01 vs. LPS WT; # *p* < 0.05 vs. LPS α7 nAChR KO; $ *p* < 0.05 vs. LPS + Melatonin WT. Two-way ANOVA with Tukey’s multiple comparisons test. Data of all experiments are represented as mean ± SD.

As a consequence of all this accumulated in vitro evidence where melatonin reduced IL-1β release, inflammasome components expression, intracellular ROS and nitrites production, this research should be continued in animal models to confirm these initial results. Therefore, the next step was to evaluate the effects of melatonin in WT and α7 nAChRs KO mice. First, we used the Novel Object Recognition (NOR) test, a widespread behavioral assay to determine memory in mice [37], to evaluate whether melatonin is able to restore the cognitive deficits induced by LPS. The protocol used is described in Figure 6A. The discrimination index showed that non-treated animals (sham group) had a value of around 0.5 in WT and α7 nAChRs KO mice, which means a preference for the new object. In contrast, LPS-injected animals (250 µg/kg, i.p.) of both groups obtained a significant and similar reduction in the discrimination index, indicative of memory impairment (Figure 6B). In WT mice, pretreatment of 24 h with melatonin (10 mg/kg, i.p.) was able to significantly increase the discrimination index value; however, melatonin was not able to improve the memory deficits provoked by LPS in α7 nAChRs KO mice (Figure 6B). After the behavioral test, animals were weighed, and percentage of total weight loss was calculated (compared to the weight before LPS injection). LPS produced a weight loss between 6.8% and 8% in both groups, while melatonin did not modify these values; furthermore, we did not detect differences between WT and α7 nAChRs KO groups (Figure S1A). Furthermore, to control that LPS did not produce a reduction in animal mobility that could affect to discrimination index results, we calculated the total exploring time of each animal group. We did not observe differences among groups, which means that LPS at the used dose (250 µg/kg, i.p.) did not alter the mice exploring behavior (Figure S1B). 

**Figure 7:**
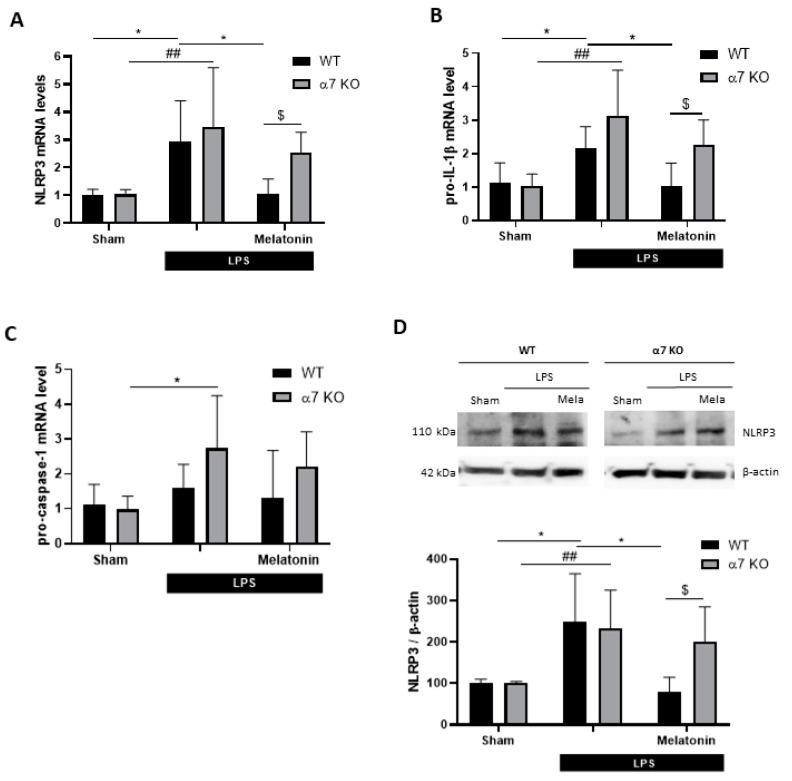
Expression of genes and proteins of the inflammasome complex in the hippocampus of WT and α7 nAChR KO mice subjected to NOR test (see protocol in Figure 6A). mRNA levels of (**A**) NLRP3, (**B**) pro-IL-1β and (**C**) pro-caspase-1 genes in hippocampus of WT (*n* = 7–8) and α7 nAChR KO (*n* = 5–6) mice after LPS (250 μg/kg) and/or melatonin (10 mg/kg) treatment. (**D**) Representative immunoblot and quantification of NLRP3 protein expression in hippocampus of WT (*n* = 7–8) and α7 nAChR KO (*n* = 5–7) mice. * *p* < 0.05 vs. LPS WT; ## *p* < 0.01 vs. LPS α7 nAChR KO; $ *p* < 0.05 vs. LPS + Melatonin WT. Two-way ANOVA test with Tukey’s multiple comparisons test. Data of all experiments are represented as mean ± SD.

Mice subjected to NOR were euthanized immediately after the test, the brains were gently removed and the hippocampi were dissected. The hippocampi were used to analyze inflammasome genes and protein expression by qRT-PCR and Western blot, respectively. In WT mice, we found that LPS (250 µg/kg, i.p.) produced a significant increase in NLRP3 and pro-IL-1β gene expression (between 2- and 3-fold). In both cases, melatonin treatment (10 mg/kg, i.p.) reduced their expression (Figure 7A,B). As occurred in the BV2 culture model, neither LPS nor melatonin modified pro-caspase-1 gene expression (Figure 7C). In α7 nAChRs KO mice, we observed that LPS produced a similar increase in NRLP3 and pro-IL-1β gene expression, whereas melatonin did not reduce the expression of any of these genes (Figure 7A,B). Furthermore, we found significantly higher gene expression levels in α7 nAChRs KO melatonin-treated mice compared to melatonin-treated WT mice. Regarding protein expression, Western blot revealed that NLRP3 is increased by 150% in both LPS-treated groups compared to the sham. Melatonin lowered NLRP3 protein to sham levels in WT mice, while in α7 nAChRs KO melatonin-treated animals had significantly elevated NLRP3 expression compared to melatonin-treated WT group (Figure 7D).

**Figure 8:**
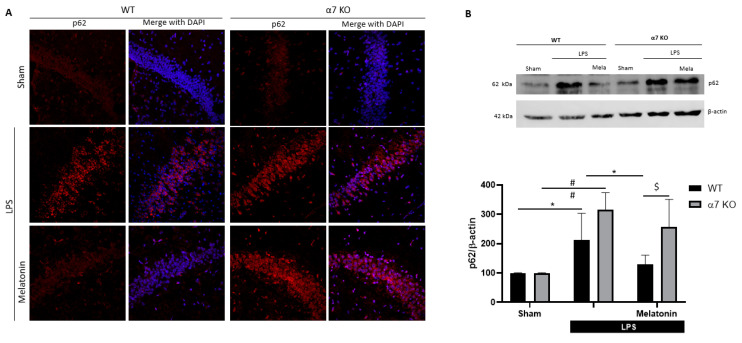
Autophagic marker p62 in the hippocampus of WT and α7 nAChR KO mice after LPS and melatonin treatments as described in Figure 6A. (**A**) p62 immunostaining of CA1 region of the hippocampus of mice injected with melatonin (10 mg/kg) followed or not by LPS (250 µg/kg) after 24h. Original magnifications for all images, 40×. (**B**) Representative immunoblot and quantification of p62 protein expression analyzed by Western Blot in the hippocampus of WT and α7 nAChR KO mice. * *p* < 0.05 vs. LPS WT; # *p* < 0.05 vs. LPS α7 nAChR KO; $ *p* < 0.05 vs. LPS + Melatonin WT. Two-way ANOVA test with Tukey’s multiple comparisons test. Data of all experiments are represented as mean ± SD.

Finally, we assessed autophagy in vivo through the detection of p62. Immunofluorescence images of hippocampal CA1 regions revealed an increase in p62 staining in both LPS groups (Figure 8A). Melatonin reduced p62 immunodetection in WT mice, whereas no change was observed in α7 nAChRs KO mice (Figure 8A). The analysis of p62 protein expression by Western blot confirmed the results observed by immunofluorescence. LPS induced a significant increase of p62 that was reverted by melatonin in WT animals, and this effect was not presented in α7 nAChRs KO mice (Figure 8B). These results obtained in vivo, using WT and α7 nAChRs KO mice, indicate that α7 nAChRs play a key role in melatonin-mediated effects.

## 4. Discussion

**Figure 9:**
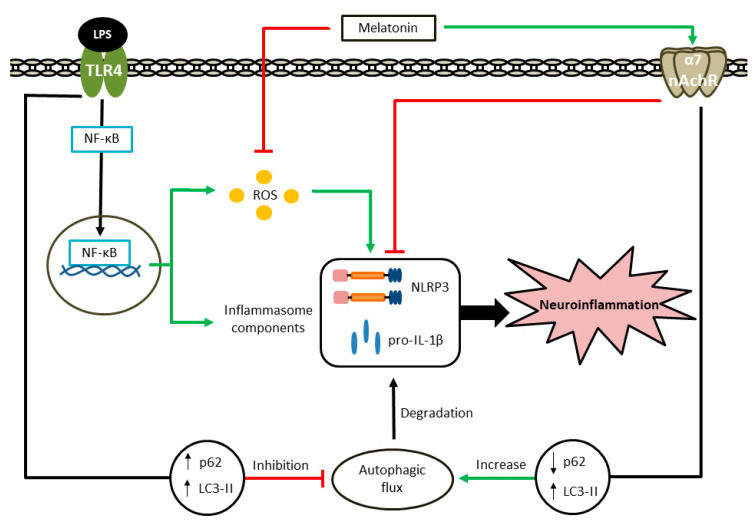
Illustration showing melatonin actions that reduce NLRP3 inflammasome expression. LPS activates TLR4 receptor that leads to an increase of ROS production and NLRP3 inflammasome components expression. Moreover, p62 and LC3-II proteins are raised, which means that autophagic flux is inhibited. As a result, NLRP3 and pro-IL-1β accumulate in microglia. On the other hand, melatonin acts as a scavenger of ROS and activates α7 nAchRs. This activation reduces inflammasome components expression. In addition, it increases the autophagic flux, which promotes the degradation of inflammasome proteins. Consequently, melatonin contributes to alleviate neuroinflammation.

In the present study, we provide new insights on the protective role of melatonin against LPS-induced neuroinflammation and oxidative stress modulated through α7 nAChRs activation both in vitro and in vivo. We found that LPS-induced inflammation leads to an increase in NLRP3 inflammasome protein due to an autophagic flux impairment. In this regard, melatonin has a potent anti-inflammatory effect by restoring the LPS-induced autophagic flux blockage. The determining factors by which melatonin reduces NLRP3 inflammasome activation include: (i) α7 nAChRs activation, (ii) reduction of inflammasome components expression (NLRP3, pro-IL-1β) and (iii) decrease of ROS production. Taken together, these evidences document that restoration of microglial autophagic flux may be a promising starting point in the development of more effective therapies for alleviating neuroinflammation (Figure 9).

Autophagy is a cellular process evolutionarily conserved that allows continuous delivery of damaged proteins and organelles to the lysosome for its degradation. In the last years, this process has been proposed to be central for the maintenance of cell homeostasis in both physiological and pathological situations. It has been reported that a lack of basal autophagy in the brain is common in different neurodegenerative diseases. In Alzheimer’s disease models, there is an increased inflammatory reaction following the disruption of microglial autophagy induced by β-amyloid treatment [9]. It has been reported that LPS induces neuroinflammation in microglia by activating the mTOR pathway, inhibiting autophagosome formation [39]. In the same direction, our results show that LPS inhibits autophagic flux by increasing LC3-II and p62, similarly to the well-known autophagy inhibitor chloroquine. In addition, we obtained the same results both in vitro (in BV2 and in mice primary cultures) and in vivo (in LPS-treated mice). In the case of immune response, there is a strong relationship between autophagy and inflammation. Regarding this issue, the role of autophagy in modulating inflammation is gaining attention and new signaling routes are emerging.

It is well-known that autophagy negatively regulates the activation of the NLRP3 inflammasome, thereby inhibiting the inflammatory response of the body and reducing the inflammatory injury of tissues in response to diseases. Here we show that the inhibition of autophagy, both with LPS and chloroquine, induced NLRP3 inflammasome activation. We observed an increase in mRNA and protein expression of the inflammasome components, probably mediated by NF-κB as previously described [42,43]. Hence, NLRP3 and autophagy pathways are linked by reciprocal regulation, denoting the potential anti-inflammatory role of the maintenance of correct autophagic flux. Furthermore, the treatment of cells with melatonin enhance autophagy by increasing LC3-II and reducing p62, in the same way that the autophagy-inductor rapamycin. We think that NLRP3 inflammasome activation is mainly taking place in microglial cells, since microglia are the resident macrophages of the brain and as such are described to secrete IL-1β. Astrocytes, the most abundant cell type in the brain, could also participate in this process. However, it has recently shown that astrocytes only express caspase-1 and does not express NLRP3 nor ASC proteins [44]. Thus, it seems that astrocytes do not have functional NLRP3 inflammasomes.

Another important point to understand the role played by IL-1β liberated through NLRP3 activation in neurodegenerative diseases, is to characterize in detail the inflammasome expression and activation in CNS inflammatory cells. In the injured brain they play important roles during glial scar formation, a process referred to as reactive astrogliosis [23]. However, it is not entirely clear if astrocytes are able to produce IL-1β. In addition, there are only few studies about the role astrocytes can play during neuroinflammation regarding inflammasome activation [24]. Therefore, we aimed to investigate the relative contribution of microglia and astrocytes to inflammasome activation in the context of neurodegenerative disease-related peptide stimulation.

There is increasing evidence showing that melatonin could enhance autophagy in different disease models like traumatic brain injury, Alzheimer’s disease or prion disease, having a beneficial effect in the disease [45,46]. Accordingly, we observed that LPS induced a short-term lack of memory in mice measured using the novel object recognition test, which is related to the inhibition of autophagic flux and the increase in the inflammatory NLRP3 components. In this regard, it has been observed that melatonin treatment was able to abolish these effects induced by LPS, which indicates the importance of the preservation of autophagic flux for a rapid anti-inflammatory response, leading to the recovery of short-term memory. 

Signaling through α7 nAChR subtype is involved in a variety of biological functions such as neuroprotection, synaptic plasticity and neuronal survival; and it is considered a prominent therapeutic target in several diseases [33,47,48]. Recently, nAChRs have been shown to regulate inflammation via the α7 nAChRs activation in macrophages [48,49,50]. In previous studies, we showed that melatonin provides a protective effect in brain ischemia that, at least in part, depends on α7 nAChR activation [31]. Moreover, it has been demonstrated that melatonin could regulate autophagic flux via α7 nAChRs in prion disease [32]. In view of this evidence, we demonstrated that the activation of autophagic flux by melatonin inhibited ROS-producing NLRP3-dependent inflammasome and reduced the inflammatory response. These effects are partially mediated through α7 nAChRs, whereas in α7 nAChRs knock-out mice, melatonin did not produce any effect. 

Wessler et al. [51] demonstrated the relationship between the cholinergic system and melatonin in the pineal gland. They showed a 10-fold increase in acetylcholine content as well as ChAT enzyme activity in pineal glands during the night period. One possible explanation for the α7 nAChR-mediated anti-inflammatory effect of melatonin is that it may be increasing the production of ACh expression, which could activate mAChRs and nAChRs in immune cells, inducing a potent anti-inflammatory effect. However, this hypothesis needs to be further explored, since immune cells possess all the required components to constitute an independent cholinergic system, including ChAT and acetylcholinesterase (AChE) as well as mAChRs and nAChRs [52]. Furthermore, recent findings suggest that ACh synthesized by immune cells plays an essential role in the regulation of immune function by triggering signals that initiate and terminate cytokine production in immune cells [53]. 

In conclusion, we have shown that melatonin: (i) displayed a potent anti-inflammatory response, (ii) inhibited ROS-producing NLRP3-dependent inflammasome (iii) enhanced autophagic flux, and (iv) these effects are partially mediated through autophagy activation via α7 nAChR activation, which supports the hypothesis that melatonin may modulate the complex interplay of α7 nAChR and autophagy signaling.

## Supplementary Materials

The following are available online at https://www.mdpi.com/2076-3921/9/12/1299/s1, Figure S1. Loss of body weight and mobility of animals subjected to NOR test.
