# Supplementary material for: Melatonin Reduces NLRP3 Inflammasome Activation by Increasing α7 nAChR-Mediated Autophagic Flux
Source: Antioxidants (Basel). 2020 Dec 18;9(12):1299. doi: 10.3390/antiox9121299 (PMC7767051; doi:10.3390/antiox9121299)
Supplement: Supplementary file 1 [file antioxidants-09-01299-s001.pdf]

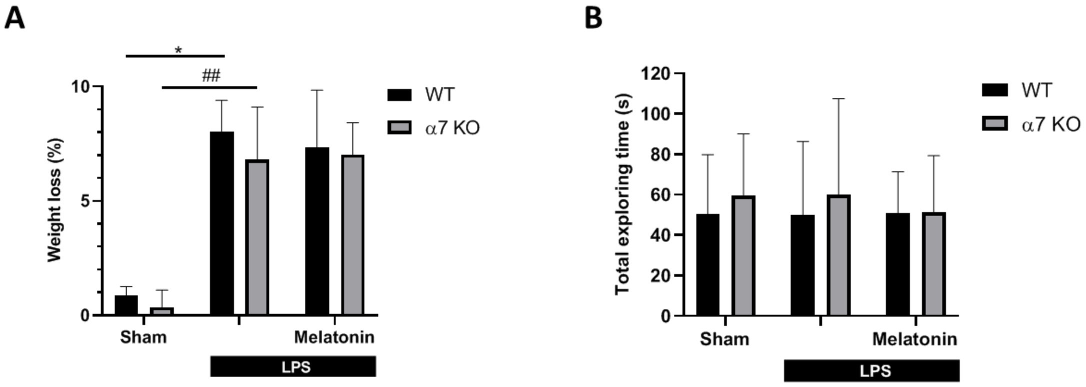

Figure S1. Loss of body weight and mobility of animals subjected to NOR test. **(A)** Animals were weighed before LPS injection and after NOR test (n = 7-8 per group). Percentage of body weight loss was calculated as follows: [(weight before injection – weight after NOR) / weight before injection x 100]. *p < 0.05 vs LPS WT and ##p < 0.01 vs LPS $\alpha 7$ nAChR KO. **(B)** Total exploring time of mice subjected to NOR test in both WT and $\alpha 7$ nAChR KO groups(n = 7-8 per group).
